# A knowledge representation approach using fuzzy cognitive maps for better navigation support in an adaptive learning system

**DOI:** 10.1186/2193-1801-2-81

**Published:** 2013-03-05

**Authors:** Konstantina Chrysafiadi, Maria Virvou

**Affiliations:** Department of Informatics, University of Piraeus, Piraeus, Greece

**Keywords:** Knowledge representation, Knowledge dependencies, Fuzzy cognitive maps

## Abstract

In this paper a knowledge representation approach of an adaptive and/or personalized tutoring system is presented. The domain knowledge should be represented in a more realistic way in order to allow the adaptive and/or personalized tutoring system to deliver the learning material to each individual learner dynamically taking into account her/his learning needs and her/his different learning pace. To succeed this, the domain knowledge representation has to depict the possible increase or decrease of the learner’s knowledge. Considering that the domain concepts that constitute the learning material are not independent from each other, the knowledge representation approach has to allow the system to recognize either the domain concepts that are already partly or completely known for a learner, or the domain concepts that s/he has forgotten, taking into account the learner’s knowledge level of the related concepts. In other words, the system should be informed about the knowledge dependencies that exist among the domain concepts of the learning material, as well as the strength on impact of each domain concept on others. Fuzzy Cognitive Maps (FCMs) seem to be an ideal way for representing graphically this kind of information. The suggested knowledge representation approach has been implemented in an e-learning adaptive system for teaching computer programming. The particular system was used by the students of a postgraduate program in the field of Informatics in the University of Piraeus and was compared with a corresponding system, in which the domain knowledge was represented using the most common used technique of network of concepts. The results of the evaluation were very encouraging.

## Background

Recent technological developments facilitate the provision of individually customized instruction to large audiences ( Akbulut and Cardak 2012 ), and lead to rapid growth of Adaptive Learning Systems (ALSs). ALSs have attracted considerable attention over the last decade, since adaptive e-learning is suitable for teaching heterogeneous student populations in higher education (Schiaffino et. al. 2008 ). The most known technology for adaptive e-learning is Intelligent Tutoring Systems (ITSs), which belong to an advanced generation of computer-based instructional systems that provide students with highly personalized learning experience, by adapting the content and its presentation to the student's needs and preferences (Jeremic et. al. 2012 ).

It is not effective to assume that all learners will have to follow the same instructional model. All learners should not be advised to read the same material and with the same order. The learning material should be delivered to learners with respect to their knowledge level and personal needs. A solution for offering adaptive navigation is the adaptive navigation support technology, which is known for its ability to help students acquire knowledge faster, improve learning outcomes and reduce navigational overhead (Brusilovsky et al. 2011 ). Many ITSs adopt the adaptive navigation support technology, which supports user navigation in hyperspace by adapting to the goals, preferences and knowledge of the individual user ( Brusilovsky 2007 ). It sustains the student in hyperspace orientation and navigation by adaptively sorting, annotating or partly hide the links that constitute the domain knowledge material, to make easier the choice of the next link to proceed ( Brusilovsky 1998 ).

An important aspect that has to be specified when applying adaptive navigation support is the links that constitute the domain knowledge, their order and the relations among them. In other words, the domain knowledge representation has to be specified. The domain knowledge module is one of the most major modules of an ITS. It contains a description of the knowledge or behaviors that represent expertise in the subject-matter domain the ITS is teaching. The particular module has been introduced in ITS but its use has been extended to most current educational software applications that aim to be adaptive and/or personalized. The domain knowledge module is responsible for the representation of the subject matter taking into account the course modules, which involve domain concepts. Either the order in which each domain concept has to be taught or the knowledge dependencies that exist between the domain concepts of the learning material have to be represented.

The domain knowledge representation is the base for the representation of the learner’s knowledge, which is usually performed as a subset of the domain knowledge. However, the representation of the learner’s knowledge is a moving target. The student’s knowledge level of a domain concept usually is affected by her/his knowledge level of other related domain concepts. For example, a new domain concept may be completely unknown to the learner but in other circumstances it may be partly known due to previous related knowledge of the learner. On the other hand, domain concepts which were previously known by the learner may be completely or partly forgotten. Hence, currently they may be partly known or completely unknown. Therefore, the knowledge dependencies that exist between the domain concepts of the learning material, as well as their “strength of impact” on each other have to be represented. A solution to this is the theory of Fuzzy Cognitive Maps (FCMs), which is used to model the behavior of complex systems (Leon et al. 2011 ). A FCM is a cognitive map within which the relations between the elements (e.g. concepts, events, project resources) of a "mental landscape" can be used to compute the "strength of impact" of these elements.

This paper presents a knowledge representation approach which uses FCMs. They are used in order to represent graphically the knowledge dependencies that exist between the domain concepts of the learning material, as well as the “strength of impact” of each domain concept on others. The particular knowledge representation approach has been implemented in an e-learning adaptive system for teaching computer programming. This system was used by the students of a postgraduate program in the field of Informatics in the University of Piraeus. This system was compared with a corresponding system, which used the most common used technique for representing the domain knowledge: a network of concepts. The results of the evaluation were very encouraging.

The remainder of this paper is organized as follows. In section 2, the related work in the domain knowledge representation and FCMs is presented. In section 3, the Fuzzy Cognitive Maps technology is presented and described. In section 4, a description of how to use Fuzzy Cognitive Maps for the representation of the knowledge domain of an adaptive learning system follows. In section 5, the implementation of Fuzzy Cognitive Maps in the domain knowledge representation of an e-learning adaptive system for teaching computer programming is presented. In section 6, a fully evaluation of the suggested knowledge representation approach is described. Finally, in section 7, the knowledge representation through FCMs was discussed and the conclusions drawn from this work are presented.

### Related work

The domain knowledge representation in an adaptive and/or personalized tutoring system is an important factor for providing adaptivity. To enable communication between system and learner at content level, the domain model of the system has to be adequate with respect to inferences and relations of domain entities with the mental domain of a human expert (Peylo et. al. 2000 ). The most common used techniques of domain knowledge representation in adaptive tutoring systems are hierarchies and networks of concepts. A hierarchical knowledge representation is usually used in order to specify the order in which the domain concepts of the learning material have to be taught ( Lin and Ruimin 2011 ; Siddara and Manjunath 2007 ; Vasandani and Govindury 1995 ), and can be implemented through trees ( Kumar 2005 ; Geng et al. 2011 ). However, hierarchies do not give information about the dependency relations that exist between the domain concepts. This kind of information is given by the network representation. Many adaptive tutoring systems, such as Web-PTV ( Tsiriga and Virvou 2003 ), DEPTHS (Jeremic et. al. 2009 ) and IDEAL (Alsubait & Khamis 2011 ) use a network of concepts for representing the domain knowledge.

In a network of concepts, nodes represent concepts and arcs represent relations between concepts. However, the relations between concepts are restricted to “part-of”, “is-a” and prerequisite relations. They do not give answers to the questions “If a student learn the concept A, which is her/his knowledge level of the depended domain concept B?”, or “If the student's knowledge of concepts A, B and C improves, how is her/his knowledge of the depended concept D affected?”, or “If the student has misconceptions on the domain concept A, how is her/his knowledge level of the depended concepts B, C and D affected?”. In other words, they do not represent how the knowledge of a domain concept of the teaching material, may be affected by the knowledge of another domain concept. Therefore, a graph, in which nodes represent the domain concepts of the learning material and the relations between them are “related-to” relations and they are accompanied with a number which declares the degree of knowledge level’s increase or decrease of the concept in relation with changes on the knowledge level of the related concepts with this, is needed.

Graph techniques have extensively used for knowledge representation. In artificial intelligence they have been introduced under the name of semantic networks, which are graphic structures used to represent concepts and knowledge in computer ( Lehmann 1992 ). Later John Sowa (1976 ) created conceptual graphs a family of semantics networks, with application to artificial intelligence, computer science and cognitive science ( Sowa 1984 ). A conceptual graph is a knowledge representation tool which helps the learning process (BinShyan et. al. 2006 ). An intelligent tutoring system can be based on conceptual graphs ( Tangjin and Xiahong 2010 ) and indeed the technique of conceptual graphs has been applied to programming tutors ( Pillay 2000 ; Smith, 2009 ). However, conceptual graphs depict mainly the logical relationships between the domain concepts. They are not sufficient for the representation of the knowledge dependencies, as they have been described above.

In view of the above, Fuzzy Cognitive Maps (FCMs) seem to be ideal for knowledge representation, since its structure resembles to this of the above desired graph. FCMs are able to incorporate experts' knowledge ( Papageorgiou and Salmeron 2012 ; Salmeron 2009 ; Salmeron et. al. 2012 ) and, also, they approach to representation of knowledge by emphasizing on the connections and the structure ( Lin 2007 ). The main reasons for using the FCM approach are (van Vliet et. al., 2010 ): easy of use, easy to construct and parameterize, flexibility in representation, low time performing, easily, understandable/transparent to non-experts and lay people (Rodriguez-Repiso et. al., 2007 ), handle with complex issues related to knowledge elicitation and management. A collection of papers with applications of FCMs in various disciplines is presented in Glykas (2010 ). Over the past two decades, FCMs have attracted wide varieties of researchers in terms of representing knowledge and artificial intelligence in engineering applications ( Aguilar 2005 ). However, the contribution of FCMs to the knowledge representation of an adaptive tutoring system has not been discussed before.

### Fuzzy cognitive maps

Fuzzy Cognitive Mapping is a combination of fuzzy logic and cognitive mapping, and it is a way to represent knowledge of systems which are characterized of uncertainty and complex processes. Fuzzy Cognitive Maps (FCMs) were introduced by Kosko (1986 ; 1992 ) and since then they have gradually emerged as a powerful paradigm for knowledge representation (Song et. al. 2012 ). They provide a more flexible and natural mechanism for knowledge representation and reasoning, which are essential to intelligent systems (Miao et. al. 2010 ). They constitute a way to represent real-world dynamic systems, in a form that corresponds closely to the way humans perceive it ( Papageorgiou 2011 ). That is the reason of their extensive use in a wide range of application (Craiger et. al. 1996 ; Kosko 1999 ; Miao and Liu 2000 ; Rodriguez-Repiso et. al. 2007 ; Stylios and Groumpos 2004 ).

**Figure 1:**
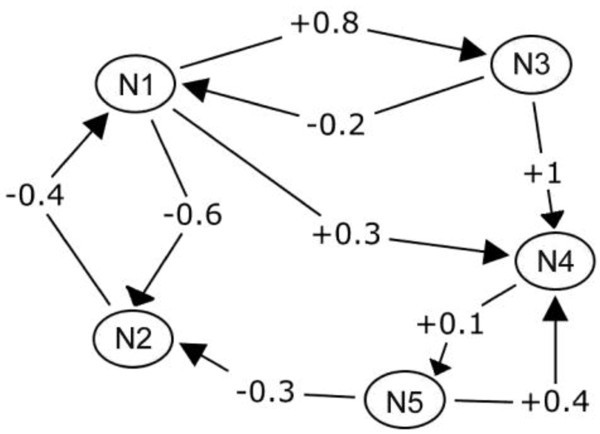
**A fuzzy cognitive map.**

A Fuzzy Cognitive Map illustrates the whole system as a combination of concepts and the various relations that exist between its concepts (Azadeh et. al. 2012 ; Song et. al. 2011 ; Stula et. al. 2010 ) (Figure 1). A FCM consists of nodes (N_1_, N_2_, … N_n_), which represent the important elements of the mapped system, and directed arcs (e_ij_), which represent the causal relationships between two nodes (N_i_, N_j_). The directed arcs are labeled with fuzzy values in the interval [-1, 1], that show the “strength of impact” between the factors. A positive value indicates a positive causality between two factors, while a negative value indicates a negative causality between two factors. In particular, lets f1 and f2 be two related factors in a FCM. The positive value on the directed arc that connect f1 with f2, means that the increase of the value of f1 leads to the increase of the value of f2, or the decrease of the value of f1leads to the decrease of the value of f2. If the value is negative, it means that the increase of the value of f1 leads to the decrease of the value of f2, or the decrease of the value of f1leads to the increase of the value of f2.

According to Stach et. al. ( 2005 ) the mathematical formulation of a FCM is the following: A FCM is a 4-tuple (N, E, C, f), where:

1. N = {N_1_, N_2_, … N_n_} is the set of n concepts forming the nodes of a graph
2. E: (N_i_, N_j_) → e_ij_ is a function of NxN to K associating e_ij_ to a pair of concepts (N_i_, N_j_), with e_ij_ denoting a weight of directed edge from N_i_ to N_j_, if i ≠ j and e_ij_ equal to zero if i = j. Thus, E(NxN) = (e_ij_)єK^nxn^ is a connection matrix.
3. C: N_i_ → C_i_ is a function that at each concept N_i_ associates the sequence of its activation degrees such as for tєN, C_i_(t)єL given its activation degree at the moment t. C(0)єL^n^ indicates the initial vector and specifies initial values of all concept nodes and C(t)єL^n^ is a state vector at certain iteration L.
4. f:R → L is a transformation function, which includes recurring relationship on t ≥ 0 between C(t + 1) and C(t).

The transformation function is used to confine the weighted sum to a certain range, which is usually set to [0, 1].

### Domain knowledge representation with FCMs

The domain knowledge representation plays an important role in the adaptation of a tutoring system. That happens because the domain knowledge module constitutes the base of either the presentation of the learning material or the representation of the student's knowledge level. An important factor that has to be taken into consideration during the process of the domain knowledge representation is that the domain concepts, which constitute the learning material, are not independent from each other. In particular, the knowledge about a domain concept may help a student to learn another domain concept or the difficulty of a student to understand a domain concept may means that s/he has misconceptions on another related domain concept. For example, a new domain concept may be completely unknown to the learner or it may be partly known due to her/his previous knowledge on another related domain concept. Furthermore, the poor performance of a learner on a domain concept may mean that s/he has forgotten related domain concepts, which were previously known. In other words, the knowledge level of a domain concept of the learning material can either increase or decrease, in some degree, the knowledge level of a depended domain concept. That is the reason for the need of the determination and representation of the knowledge dependencies that exist between the domain concepts of the learning material.

**Figure 2:**
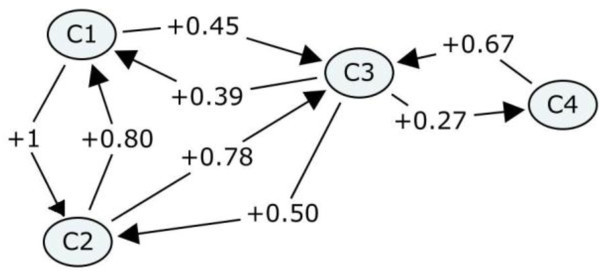
**Domain knowledge representation using a FCM.**

In view of the above, FCMs are used to represent the knowledge dependencies of the domain concepts of the learning material and the “strength of impact” of a concept on its related concepts. In a FCM, which is used for the representation of the domain knowledge of an adaptive learning system, the nodes represent the domain concepts of the learning material and the directed arcs connect those concepts whose knowledge affects each other (Figure 2). The values which are labeled on arcs of the FCM are only positive, since the increase of the knowledge level of a domain concept leads to the increase of the knowledge level of a depended domain concept, and the decrease of the knowledge level of a domain concept leads to the decrease of the knowledge level of a depended domain concept. Therefore, the values of the arcs belong to the interval (0, 1].

The arcs in the FCM which represent the domain concepts’ dependencies of the domain knowledge are bidirectional. Furthermore, the value of the arc C_i_ → C_j_ is not essentially equal to the value of the arc C_j_ → C_i_. This is happened due to the fact that changes on the knowledge level of C_i_ may affect the knowledge level of C_j_ in a different degree than changes on the knowledge level of C_j_ affect the knowledge level of C_i_. For example, in the field of algorithms, if a learner knows how to calculate an average in an iterative structure with concrete number of loops, it implies that s/he knows also how to calculate a sum in an iterative structure with no concrete number of loops, since calculating an average incorporates calculating the corresponding sum. Similarly, if a student excels at calculating a sum in an iterative structure with no concrete number of loops, it implies that s/he knows partly how to calculate an average in an iterative structure with concrete number of loops, since calculating the sum is the base for calculating an average. However, these two domain concepts do not affect each other in the same degree. The knowledge level of calculating an average in an iterative structure with concrete number of loops affects 100% (the value of the corresponding arc is 1) the knowledge level of calculating a sum in an iterative structure with no concrete number of loops, while the knowledge level of calculating a sum in an iterative structure with no concrete number of loops affects 80% (the value of the corresponding arc is 0.8) the knowledge level of calculating an average in an iterative structure with concrete number of loops. These “strengths of impact” have been defined by experts in the field of algorithms and programming languages.

Thus, in a correspondence with its theoretical definition, a FCM that is used to represent the domain knowledge of the learning material is a 4-tuple (C, W, KL, f), where:

1. C = {C_1_, C_2_, … C_n_} is the set of concepts of the domain knowledge.
2. W: (C_i_, C_j_) → w_ij_ is a connection matrix, where w_ij_ is a weight of the directed ard from C_i_ to C_j_, which denotes that the knowledge level of the concept C_i_ affects that of concept C_j_.
3. KL is a function that at each concept C_i_ associates the sequence of its activation degree. In other worlds, KL_i_(t) indicates the value of a concept’s knowledge level at the moment t.
4. f is a transformation function. For the definition of the transformation function the following limitation has to be taken into account. The knowledge level of a domain concept is affected, each time, only by the knowledge level of the most recently read concept. The reason for this is the fact that the learner’s knowledge level is affected either by the new knowledge that s/he has obtained, or by the knowledge that s/he has forgotten, each time. Consequently, the KL value of a concept is affected only by the KL value of the most recently read concept, regarding the weight of the directed arc that connects them. Therefore, the transformation function for a FCM, which is used to represent the domain knowledge of the learning material, is defined as: KL_i_(t + 1) = f(KL_i_(t) ± w_ji_*p_j_*KL_i_(t)/100), where p_j_ is the percentage of the difference on the value of the knowledge level of the most recently read concept C_j_, with p_i_ = (KL_j_(t + 1)-KL_j_(t))*100/KL_j_(t). Also, the + is used in case of increase and the – is used in case of decrease.

It must be referred that the initial values KL(0) for concepts are zero. The reason for this is the fact that a learner is considered as novice in the beginning of the learning process. The changes on these values indicate the progress or no-progress of the learner.

For a better computation of the knowledge dependencies, the connection matrix (w-matrix) of the FCM is used. This matrix depicts the “strength of impact” between the concepts of the learning material. It represents the weight of the directed arcs of FCM in a more simple and clear way. For example, Table 1 is the w-matrix of the FCM that is depicted in Figure 2. The number of rows of this matrix is equal to the number of columns, and it is also equal to the number of the nodes (domain concepts) that are depicted to the FCM. The values of the directed arcs of the FCM are written into the cells of the matrix. The matrix is completed row by row. The value of the “strength of impact” of the domain concept that corresponds to the matrix’s row i on the domain concept that corresponds to the matrix’s column j is written into the matrix's cell (i, j). For example, the value of the “strength of impact” of the domain concept C_1_ on the domain concept C_3_, which are depicted in Figure 2, is written in the corresponding matrix's cell (1, 3) (Table 1). The values of the matrix’s main diagonal are zero, since changes on the knowledge of a domain concept cannot affect the domain concept itself. This confronts to the FCM definition (Section 3), which denotes that e_ij_ = 0 if i = j.

**Table 1:** **W-matrix with knowledge dependencies**

	C_1_	C_2_	C_3_	C_4_
**C**_**1**_	0	1	0.45	0
**C**_**2**_	0.80	0	0.78	0
**C**_**3**_	0.39	0.50	0	0.27
**C**_**4**_	0	0	0.67	0

If a row of the matrix is read, then information about the domain concepts that are affected by the concepts which corresponds to the particular row, as well as about its “strength of impact” on them, will be extracted. For example, if the second row of the matrix that is depicted in Table 1 is read, then information about the fact that changes on the knowledge level of the domain concept C_2_ affects at 80% the knowledge level of the domain concept C_1_ and at 78% the knowledge level of the domain concept C_3_, will be extracted. If a column of the matrix is read, then information about the domain concepts that affect the concept which corresponds to the particular column, as well as about the “strength of impact” of them on it will be obtained. For example, if the fourth column of the matrix that is depicted in Table 1 is read, then information about the fact that the knowledge level of the domain concept C_4_ is affected only by the changes on the knowledge of the domain concept C_3_, will be obtained.

According to the matrix that is depicted in Table 1, if the knowledge level of the domain concept C_3_ is increased, then the knowledge level of the domain concepts C_1,_ C_2_ and C_4_ will be increased also, but not in the same degree. According to the transformation function of the FCM the following will be happened: KL_1_(t + 1) = KL_1_(t) + 0.39*p_3_*KL_1_(t)/100, KL_2_(t + 1) = KL_2_(t) + 0.5*p_3_*KL_2_(t)/100, and KL_4_(t + 1) = KL_4_(t) + 0.27* p_3_*KL_4_(t)/100. In particular, if the knowledge level of C_3_ is increased 100%, then the increase of the knowledge level of C_1_, C_2_ and C_4_ will be 39%, 50% and 27% correspondingly. While, if it is increased 50%, then the increase of the knowledge level of C_1_, C_2_ and C_4_ will be 19.5%, 25%, and 13.5% correspondingly. Likewise, if the knowledge level of the domain concept C_4_ is decreased, then the knowledge level of the domain concept C_3_ will be decreased also. According to the transformation function the knowledge level of C_3_ will be decreased as KL_3_(t + 1) = KL_3_(t)-0.67*p_4_*KL_3_(t). Specifically, if the knowledge level of C_4_ is decreased 30%, then the knowledge level of C_3_ will be decreased 20.1%. It is remarkable to refer that the decrease on the knowledge level of C_3_ will cause decrease on the knowledge level of its related domain concepts C_1_ and C_2_ (C_4_ will not be decreased again because this is the domain concept that causes all the changes on the knowledge level of the remains domain concepts).

### Implementation of FCMs in a computer programming tutor

**Figure 3:**
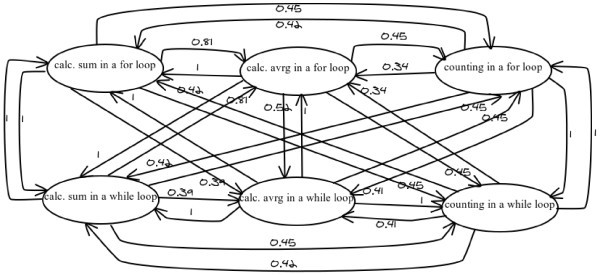
**Knowledge dependencies among the concepts.**

The knowledge representation technique that is presented in this paper has been implemented in an e-learning environment for individualized instruction on the domain of programming languages (Chrysafiadi and Virvou, 2010 ), leading to a new improved version of the particular computer programming tutor. The domain knowledge of the e-learning tutor was organized into a hierarchical structure in combination with FCMs. The FCMs were used in order to represent the dependencies that exist among the knowledge level of the domain concepts of the domain of computer programming. More specifically, they were used in order to represent the knowledge dependencies that exist among the programming structures and methodologies, as Figure 3 depicts.

The need for representation of the knowledge dependencies of the domain concepts of the domain knowledge of computer programming is crucial, since there are many programming languages, which met different needs, and learners can vary from novice to those who know some other programming language and therefore the basic principles of computer programming. Consequently, each learner has her/his own learning pace and the educational environment have to adapt to this. Evidently, a novice programmer has to read much more domain concepts of the learning material than a programmer, who already knows the principles and the basic structures of computer programming. Furthermore, the knowledge of a programming methodology (i.e. calculating sum in a for loop) may mean that the learner does not need to read another resemble programming methodology (i.e. counting in a for loop), or the knowledge of a programming structure (i.e. one-dimensional arrays) may mean that the learner can understand more easily another programming structure (multidimensional arrays), so the latter should not be considered completely unknown for the learner. Also, if the performance of a learner on a domain concept is poor, then it may mean that s/he has forgotten another relevant domain concept. For example, if a learner has difficulties in calculating a sum in a while loop, it means that her/his knowledge on the previous domain concept of “calculating a sum in a for loop” has to be decreased.

According to the presented methodology, the corresponding matrix, which “interprets” the information that is incorporated in the FCM of the Figure 3, is the following (Table 2).

**Table 2:** **W-matrix with knowledge dependencies among the programming structures**

	Calc. sum in a for loop	Calc. avrg in a for loop	Counting in a for loop	Calc. sum in a while loop	Calc. avrg in a while loop	Counting in a while loop
**Calc. sum in a for loop**	0	0.81	0.45	1	0.39	0.45
**Calc. avrg in a for loop**	1	0	0.45	1	0.52	0.45
**Counting in a for loop**	0.42	0.34	0	0.42	0.41	1
**Calc. sum in a while loop**	1	0.81	0.45	0	0.39	0.45
**Calc. avrg in a while loop**	1	1	0.45	1	0	1
**Counting in a while loop**	0.42	0.34	1	0.42	0.41	0

The knowledge dependencies and the values of them have been defined by experts of the domain knowledge of computer programming. In particular, ten professors of computer programming, whose experience counts twelve years at least, were asked to define, empirically the dependencies between the concepts of the domain knowledge of programming, as well as their “strength of impact” on each other. In particular, the concepts of the learning material were given to them and they asked to depict the concepts’ interdependencies and their weights. The mean that were given by the ten domain experts are the values which are labeled on the directed arcs of the FCM. It has to be made clear that the value 1 of a knowledge dependency does not mean that the two dependent concepts are the same. It indicates that if a learner knows the one concept, the s/he will know also the other domain concept. For example, if a learner has been tested and found to have known the “for” loop and the “while” loop and this learner knows how to calculate sum in a “for” loop, then s/he will also know how to calculate sum in a “while” loop, since the methodology is the same. That is the reason for the value 1 of the “strength of impact” of the domain concept “calculating sum in a ‘for’ loop” on the domain concept “calculating sum in a ‘while’ loop”.

Initially, the knowledge level values of all the concepts for a student are zero, as s/he is considered as novice. During the learning process s/he reads the concepts and is examined in them. In this way, the values of concepts change. However, concerning the FCM, the alterations of the knowledge level value of a concept causes change on the knowledge level value of its related concepts. Increase of the value of a concept causes increase of the value of its related concepts or decrease of the value of a concept causes decrease on the value of its related concepts. These alterations are conducted according to the transformation function of the FCM, and indicate the progress or no-progress of the learner. For example, according to the matrix that is depicted in Table 2, if the knowledge level of the domain concept “calculating sum in a ‘for’ loop” for a learner is increased at x%, then her/his knowledge level of all the related concepts with this will be increased as it is presented in Table 3. For example, if x equals to 85%, then the knowledge level of the related concepts will become as it is depicted in Table 4.

**Table 3:** **Increase on knowledge level of the depended concepts**

Domain concept	Increase (%) w_ij_*p_j_
Calc. avrg in a for loop	0.81*x
Counting in a for loop	0.45*x
Calc. sum in a while loop	1*x
Calc. avrg in a while loop	0.39*x
Counting in a while loop	0.4545*x

**Table 4:** **Increase on knowledge level of the depended concepts (x = 85)**

Domain concept	Increase (%) w_ij_*p_j_	Knowledge level KL_i_(t) (moment t)	Knowledge level (moment t + 1) KL_i_(t + 1) = KL_i_(t) + w_ji_*p_j_*KL_i_(t)/100
Calc. avrg in a for loop	68.85	68%	100%
Counting in a for loop	38.25	52%	71.89%
Calc. sum in a while loop	85	60%	100%
Calc. avrg in a while loop	33.15	58%	77.23%
Counting in a while loop	38.25	52%	71.89%

Similarly, if the knowledge level of the domain concept “calculating average in a while loop” for a learner is decreased at a percentage of ×%, then her/his knowledge level of all the related concepts with this concept will be decreased as it is presented in Table 5. For example, if × equals to 42%, then the knowledge level of the related concepts will become as it is depicted in Table 6.

**Table 5:** **Decrease on knowledge level of the depended concepts**

Domain concept	Decrease (%) w_ij_*p_j_
Calc. sum in a for loop	1*x
Calc. avrg in a for loop	1*x
Counting in a for loop	0.45*x
Calc. sum in a while loop	1*x
Counting in a while loop	1*x

**Table 6:** **Decrease on knowledge level of the depended concepts (x = 42)**

Domain concept	Decrease (%) w_ij_*p_j_	Knowledge level KL_i_(t) (moment t)	Knowledge level (moment t + 1) KL_i_(t + 1) = KL_i_(t)-w_ji_*p_j_*KL_i_(t)/100
Calc. sum in a for loop	42	68%	39.44%
Calc. avrg in a for loop	42	52%	30.16%
Counting in a for loop	18.9	60%	48.66%
Calc. sum in a while loop	42	58%	33.64%
Counting in a while loop	42	52%	30.16%

### Evaluation

In order to provide the evidence that the proposed approach is of potential value, evaluation is required. An evaluation offers information to make decision about using the product or not ( Phillips and Gilding 2003 ). In Intelligent Tutoring Systems community, the common practice of evaluation is empirical approaches ( Aimeur and Frasson 2000 ; Jeremić et. al. 2009 ; Weber and Specht 1997 ). Empirical evaluations refer to the appraisal of a theory by observation in experiments (Mulwa et. al. 2011 ).

Therefore, an experiment was conducted, in order to compare the effectiveness of the navigation support that is offered by two different systems: LeCP-A and LeCP-B. Both systems are adaptive tutors of computer programming. They intend to teach learners either the principles and structures of the computer programming, or the logic of programming and algorithms including calculating sums, averages and maximums or minimums. To this end, the learning material of both systems is broken down into 31 domain concepts that are listed in Table 7. The learner meets these concepts in sequence.

**Table 7:** **The learning material**

1. Basics	1.1. constants & variables	5. Iteration Structure Unknown no of loops	5.1. while statement
	1.2. assignment statement		5.2. calculating sum in a while loop
	1.3. arithmetic operators		5.3. counting in a while loop
	1.4. comparative operators		5.4. calculating avgr in a while loop
	1.5. logical operators		5.5. calculating max/min in a while loop
	1.6. mathematical functions		5.6. do…while statement
	1.7. input–output statements		
2. Sequence structure	2.1. a simple program structure	6. Arrays	6.1 one-dimensional arrays
			6.2. searching
3. Conditional Structures	3.1. if statement		6.3. sorting
	3.2. if…else if		6.4. two-dimensional arrays
	3.2.1 methodology of finding max/min		
	3.3. nested if		6.5. processing per row
			6.6. processing per column
4. Iteration Structure Concrete no of loops	4.1. for statement		6.7. processing of diagonals
	4.2. calculating sum in a for loop	7. Sub-programming	7.1. functions
	4.3. counting in a for loop		
	4.4. calculating avgr in a for loop		
	4.5. calculating max/min in a for loop		

**Figure 4:**
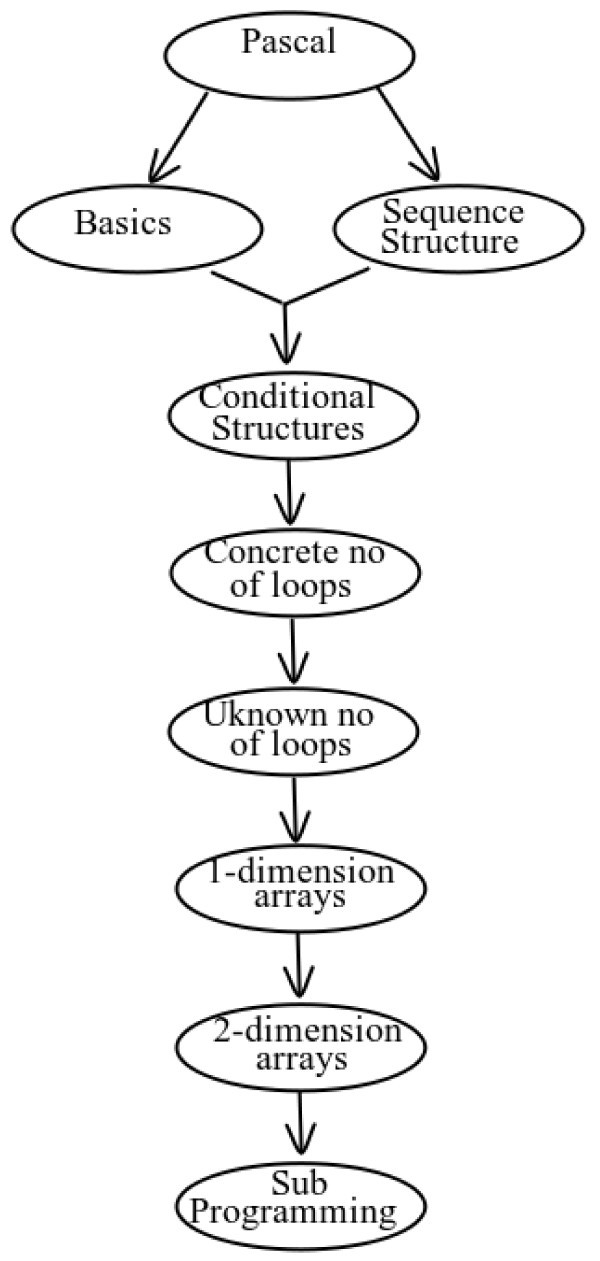
**Hierarchical structure.**

**Figure 5:**
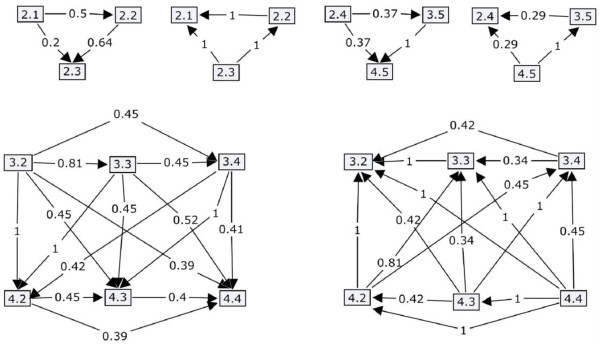
**Fuzzy cognitive maps.**

**Figure 6:**
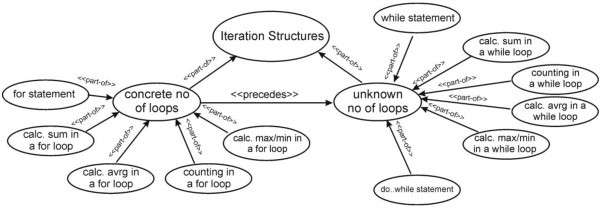
**Network of concepts.**

A difference between the two tutors is the knowledge representation technique. In particular, the domain knowledge of LeCP-A is organized into a hierarchical structure in combination with FCMs. The hierarchical structure (Figure 4) depicts the difficulty levels of the domain concepts and the order in which each topic must be taught. The FCMs (Figure 5) represent the dependency relations between the domain concepts of the learning material, concerning the influences of the knowledge level of a concept to the knowledge level of another related concept. On the other hand, LeCP-B uses a network of concepts in order to represent the structure of the learning material and the relations between its domain concepts (Figure 6). A network of concepts is a common used technique for representing the domain knowledge with nodes denoting concepts and arcs denoting relations between concepts (Tsiriga & Virvou, 2003 ; Jeremic et, al., 2009 ). However, in a network of concepts the relations between concepts are restricted to “part-of”, “is-a” and prerequisite relations. They do not depict how the knowledge level of a domain concept is affected by the knowledge level of another concept. Therefore, in LeCP-B’s network of concept, the relations are restricted to prerequisite and “part-of” relations.

In both systems, each time the learner completes a domain concept, s/he takes a test to check her/his knowledge level and progress. The results of the test determine the value of the learner’s knowledge level of the concept. In LeCP-A this value affects the knowledge level of the related concepts with this concept, regarding the FCM. However, this does not happened in LeCP-B, since in this system the knowledge level of the concepts are depicted independent from each other. For example, at the moment t, Mike is reading the concept C_3.2_ and is taking the corresponding test. He is scoring 91% at the test. The previous score of Mike in the particular concept was 70%. So, his knowledge level of the particular concept has been increased at 30%.According to LeCP-A, Mike’s knowledge level will become as it is presented in Table 8 (in column LeCP-A), while according to LeCP-B, Mike’s knowledge level will become as it is depicted in the same table in the column “LeCP-B”. In LeCP-A, the student’s knowledge level is not determined only by the test’s score, as it happens in LeCP-B, but also it is determined by the knowledge dependencies that exist among the concepts of the learning material. Therefore, LeCP-A can consider a concept as already learned for a student, monitoring her/his knowledge level of its related concepts. Furthermore, LeCP-A recognizes the domain concepts that a learner has forgotten, concerning the lack of knowledge on other related concepts. Consequently, a learner who uses LeCP-A is advised to read a concept no one, one, or more times. On the other hand, a learner who uses LeCP-B is advised to read a concept at least one time.

**Table 8:** **The knowledge level of Mike**

Domain concepts	Mike’s knowledge level		
	Moment t	LeCP-A (moment t + 1)	LeCP-B (moment t + 1)
1.1 constants & variables	100%	100%	100%
1.2 assignment statement	92%	92%	92%
1.3 arithmetic operators	100%	100%	100%
1.4 comparative operators	92%	92%	92%
1.5 logical operators	100%	100%	100%
1.6. mathematic functions	100%	100%	100%
1.7 input–output statements	100%	100%	100%
1.8 a simple program’s structure	100%	100%	100%
2.1 if statement	85%	85%	85%
2.2 if…else if	85%	85%	85%
2.3 nested if statement	65%	65%	65%
2.4 finding max, min	100%	100%	100%
3.1 for statement	100%	100%	100%
**3.2 calc. sum in a for loop**	**91**%	**91**%	**91**%
**3.3 calc. avrg in a for loop**	**50**%	**62.15**%	**50**%
**3.4 counting in a for loop**	**100**%	**100**%	**100**%
3.5 calc. max, min in a for loop	63%	63%	63%
4.1 while statement	80%	80%	80%
**4.2 calc. sum in a while loop**	**70**%	**91**%	**70**%
**4.3 counting in a while loop**	**100**%	**100**%	**100**%
**4.4 calc. avrg in a while loop**	**72**%	**80.42**%	**72**%
4.5 calc. max, min in a while loop	37%	37%	37%
4.6 do…until	75%	75%	75%
5.1 one-dimension arrays	0%	0%	0%
5.2 searching	0%	0%	0%
5.3 sorting	0%	0%	0%
6.1 two-dimensions arrays	0%	0%	0%
6.2 processing per rows	0%	0%	0%
6.3 processing per column	0%	0%	0%
6.4 processing of diagonals	0%	0%	0%
7.1 procedures	0%	0%	0%
7.2 functions	0%	0%	0%

The criterion for the evaluation of the knowledge representation technique through FCMs is the mean number of times that a learner is advised to read a domain concept, until it is considered as learned. The fewer the times are, the better navigation support is provided. For the needs of the experiment, in both systems a domain concept is considered as learned when its knowledge level is equal to or greater than 80%. Otherwise, the learner is advised to repeat the particular concept. A group of 50 students of a postgraduate program in the field of informatics at the University of Piraeus (group A) used LeCP-A in order to learn computer programming, and a group of 36 students of the same postgraduate program (group B) used LeCP-B for the same purpose. The results of the experiment showed that the mean number of reading times for group A is significantly lower than the corresponding mean for group B (Table 9), even though students of group A were advised to repeat some domain concepts due to their insufficient performance on related concepts. That means that FCMs help the system to provide more efficient navigation support.

**Table 9:** **Mean numbers of reading times**

	Group	N	Mean	Std. deviation	Std. error mean
**No_of_reading**	A	50	1.0648	.54842	.07756
	B	36	2.1686	.58693	.09782

However, how can we be sure that the different average scores are not occurred by chance, or due to differences on the education, knowledge level and abilities of the learners of the two groups, or due to the different amount of participants in the two groups? In order to test whether the different average scores of the two groups represents a real difference between the two groups, the statistical method of “Independent-sample *T*-test” was used (Carver and Nash, 2009 ). The particular statistical method used the Levene’s test, according to which, if the value of “sig.” variable is higher than 0.05, then the two variances are approximately equal, and if the value of “sig. (2-tailed)” variable is equal or lower than 0.05, then the differences between the means are statistically significant.

The results of the test are depicted in Table 10. They showed that the value of the variable “sig.” is 0.141, which is higher that 0.05, and the value of the variable “sig. (2-tailed)” is 0, which is lower than 0.05. Consequently, the difference of the means of the two groups is not occurred due to chance and, also, they are statistically significant.

**Table 10:** **Results of Levene’s test for equality of variances**

		F	Sig.	t	df	Sig. (2-tailed)
**no_of readings**	**Equal variances assumed**	2.206	.141	-8.941	84	.000

## Conclusions & discussion

The target of this paper was to present a domain knowledge representation method that can contribute to the improvement of the navigation support that an adaptive learning system provides. More specifically, the progress or no-progress of a learner indicates the need for omission or repetition of some domain concepts. If a learner excels at a domain concept, it implies that s/he does not need to read some other relative domain concepts or that a depended domain concept is already known for her/his at some degree. Furthermore, if a learner has misconceptions on a domain concept, it implies that s/he has to revise a prerequisite relative domain concept. Consequently, the aim was to represent the knowledge dependencies among the domain concepts that constitute the learning material of an adaptive learning system, as well as the “strength of impact” of them on each other. This can be done through Fuzzy Cognitive Maps.

A limitation of this approach is that the success of the FCM’s design is depended significantly on the knowledge and experience of domain experts. In particular, the nodes of the FCM, which represent the domain concepts of the learning material, are defined by domain experts. Also, the contribution of domain experts is significant for the definition of the knowledge dependencies between the domain concepts of the learning material and their strength of impact on each other. In other words, they define the values of the w-matrix of the FCM.

The presented knowledge representation approach was compared with the most common used technique for representing the domain knowledge, which is called network of concepts technique. The evaluation results showed that the presented knowledge representation approach improves the efficiency of the system’s navigation support. It recognizes either the domain concepts that are already partly or completely known for a learner or the domain concepts that s/he has forgotten, taking into account the learner’s knowledge level of the related concepts of the learning material. As a result, the presented knowledge representation approach constitutes an ideal way for representing the domain knowledge of an adaptive and/or personalized tutoring system in a more realistic way. It constitutes a driver for an adaptive and/or personalized system for delivering the learning material to each individual learner dynamically, taking into account her/his learning needs and her/his different learning pace.

However, the system’s adaptivity is depended not only on the domain knowledge representation technique, but also on the student modeling approach. The domain knowledge representation has to be combined with a well-designed student model, which will be is responsible for how the system will utilize the information which is included in the domain knowledge module, in order to make the right decisions for offering personalized instruction and support. The modeling of the learner’s knowledge level, however, depends on the way of the knowledge domain representation. So, choosing a good technique for the representation of the knowledge domain plays a significant role to the effectiveness of system’s adaptivity. Consequently, the ability of the presented knowledge representation approach to depict the possible increase or decrease of the learner’s knowledge constitutes the particular approach as a novel driver for the adaptive and/or personalized tutoring systems for providing personalized presentation of the learning material.

## Authors’ information

Konstantina Chrysafiadi born in Athens, Greece, the year 1981. She received a B.S. degree in computer science from the University of Piraeus in Greece, and a M.S. degree in information systems from the Athens University of Economics and Business. She is currently preparing her Ph.D. thesis in the Department of Informatics of the University of Piraeus, Greece. She spent three years as a teacher of informatics in a private educational institution, and five years as administrative staff in the Ministry of Education, Greece. She is currently a teacher of computer science in secondary schools in East Attica, Greece. Her fields of interest include e-learning, student modeling and teaching of programming.

Maria Virvou was born in Athens, Greece. She received a B.Sc. degree in mathematics from the University of Athens, Greece, a M.Sc. degree in computer science from the University of London (University College London), U.K. and a Ph.D. degree in computer science and artificial intelligence from the University of Sussex, U.K. She is a full professor of software engineering, Department Head and Director of Postgraduate Studes in the DEPARTMENT HEAD in the Department of Informatics, University of Piraeus, Greece. She is also Editor -in-Chief of the SpringerPlus Journal (Springer) in the area of computer science. She is also an Associate Editor of the Knowledge and Information Systems (KAIS) Journal (Springer) and a member of the editorial board of the International Journal on Computational Intelligence Studies (Inderscience). She has been the General Chair/Program Chair of over twenty (20) international conferences. She has authored three books in computer science, and over 300 published papers. She has been the principal or co- investigator on over 15 national/international research projects. She has graduated 12 Ph.D. students. Her research interests are in the area of computers and education, artificial intelligence in education, user and student modeling, e-learning and m-learning, knowledge-based software engineering and human-computer interaction.
